# *S*. Typhimurium *sseJ *gene decreases the *S*. Typhi cytotoxicity toward cultured epithelial cells

**DOI:** 10.1186/1471-2180-10-312

**Published:** 2010-12-07

**Authors:** A Nicole Trombert, Liliana Berrocal, Juan A Fuentes, Guido C Mora

**Affiliations:** 1 Laboratorio de Microbiologia, Facultad de Ciencias Biologicas y Facultad de Medicina, Universidad Andres Bello, Santiago, Chile

## Abstract

**Background:**

*Salmonella enterica *serovar Typhi and Typhimurium are closely related serovars as indicated by >96% DNA sequence identity between shared genes. Nevertheless, *S*. Typhi is a strictly human-specific pathogen causing a systemic disease, typhoid fever. In contrast, *S*. Typhimurium is a broad host range pathogen causing only a self-limited gastroenteritis in immunocompetent humans. We hypothesize that these differences have arisen because some genes are unique to each serovar either gained by horizontal gene transfer or by the loss of gene activity due to mutation, such as pseudogenes. *S*. Typhi has 5% of genes as pseudogenes, much more than *S*. Typhimurium which contains 1%. As a consequence, *S*. Typhi lacks several protein effectors implicated in invasion, proliferation and/or translocation by the type III secretion system that are fully functional proteins in *S*. Typhimurium. SseJ, one of these effectors, corresponds to an acyltransferase/lipase that participates in SCV biogenesis in human epithelial cell lines and is needed for full virulence of *S*. Typhimurium. In *S*. Typhi, *sseJ *is a pseudogene. Therefore, we suggest that *sseJ *inactivation in *S*. Typhi has an important role in the development of the systemic infection.

**Results:**

We investigated whether the *S*. Typhi *trans*-complemented with the functional *sseJ *gene from *S*. Typhimurium (STM) affects the cytotoxicity toward cultured cell lines. It was found that *S*. Typhi harbouring *sseJ_STM _*presents a similar cytotoxicity level and intracellular retention/proliferation of cultured epithelial cells (HT-29 or HEp-2) as wild type *S*. Typhimurium. These phenotypes are significantly different from wild type *S*. Typhi

**Conclusions:**

Based on our results we conclude that the mutation that inactivate the *sseJ *gene in *S*. Typhi resulted in evident changes in the behaviour of bacteria in contact with eukaryotic cells, plausibly contributing to the *S*. Typhi adaptation to the systemic infection in humans.

## Background

*Salmonella enterica *serovar Typhi (*S*. Typhi) is a human-restricted pathogen that causes enteric fever or typhoid. *Salmonella enterica *serovar Typhimurium (*S*. Typhimurium) is considered a broad host range pathogen that causes gastroenteritis in several warm-blooded animals such as calves and humans, but produces a typhoid-like systemic infection in mice [1-3]. Although the mechanism by which serovar Typhimurium causes gastroenteritis is well studied, less is known about the pathogenesis of the serovar Typhi. One limitation to the study of typhoid fever is the absence of a good animal model. For this reason, although the *S*. Typhimurium - mouse model has been used to infer *S*. Typhi important virulence mechanisms by the expression of *S*. Typhi genes in *S*. Typhimurium, the information derived from infection of mice is limited mainly because the virulence factors are tested in an heterologous system. Furthermore, *S*. Typhimurium does not cause typhoid in humans, suggesting that genetic differences between both serovars are crucial for disease development.

The evolution of a broad host pathogen, such as *S*. Typhimurium, to a host-restricted pathogen, such as *S*. Typhi, might have occurred by (i) the acquisition of genetic material through horizontal gene transfer, (ii) genome degradation (i.e., the loss of genetic information by deletion or pseudogene formation) or (iii) a combination of both of these mechanisms [4,5]. The acquisition and persistence of DNA segments containing genes with pathogenicity or virulence functions (i.e., pathogenicity islands) will depend on the advantage they confer to the pathogen infectious cycle. Thus, bacteria with a great ability to colonise different environments, such as *Pseudomonas aeruginosa*, generally have larger genomes than those that survive in restricted niches [6].

The phenomenon by which a microorganism becomes adapted to its host involves the loss of genetic functions resulting in pseudogene generation, a process termed "reductive evolution". This process has been observed in human-adapted pathogens such as *Shigella flexneri, Mycobacterium leprae *and *Salmonella *Typhi [7,8]. For example, the loss of the *ompT *gene in *Shigella *confers a virulent phenotype by allowing bacteria to transmigrate across eukaryotic cells [9,10]. In the case of *Salmonella*, some serovars have accumulated mutations that enhance their survival within their respective hosts. For example the poultry-adapted *S*. Pullorum and *S*. Gallinarum serovars are non-motile because they have a point mutation in the *flgK *gene [11,12]. When *S*. Enteritidis and *S*. Typhimurium are isolated from infected poultry, these bacteria are frequently non-motile, suggesting that the niche occupied in birds can select against flagellation [13]. These non-motile *S*. Typhimurium strains have been shown to be non-virulent when used to infect mice. Thus, in the *S. enterica*, the adaptation to a particular vertebrate host seems to drive the loss of virulence factors for some serovars. The result of this adaptation may contribute to the narrowing of the host range and to the development of host specificity [14].

*S*. Typhi is an intracellular facultative pathogen that contains over 200 pseudogenes, nearly 5% of its whole genome [15,16]. Several of the mutations that gave rise to these pseudogenes occur in systems related to pathogenicity mechanisms. For example, the *S*. Typhimurium *sseJ *gene encodes an effector protein regulated by *Salmonella *pathogenicity island 2 (SPI-2) [17,18]. SPI-2 regulated genes are related to bacterial intracellular trafficking and proliferation, and encode a protein complex known as the type III secretion system (T3SS). The T3SS mediates the injection of effector proteins from bacteria into eukaryotic cells [19-21]. These effector proteins modulate the *S*. Typhimurium endocytic pathway and allow the establishment of bacteria in a specialised vacuole termed the *Salmonella-*containing vacuole (SCV) [22]. Late stages of SCV synthesis include the formation of tubular membrane extensions known as *Salmonella-*induced filaments (Sifs). Sifs are thought to result from the fusion of late endocytic compartments with the SCV and their formation requires at least five SPI-2-dependent effectors: SifA, SseF, SseG, SopD2 and SseJ [23-26]. In this context, *S*. Typhimurium *sseJ *encodes an acyltransferase/lipase that participates in SCV biogenesis in human epithelial cell lines [25,27-29]. The coordination of SseJ and SifA is required for bacterial intracellular proliferation [30]. Some studies have shown that SseJ is needed for full virulence of *S*. Typhimurium in mice and for proliferation within human culture cells [31].

*S*. Typhi lacks several effector proteins that are crucial for the pathogenicity of the generalist serovar *S*. Typhimurium [29]. The absence of these proteins could contribute to the specificity of the human-restricted serovars, and could play a role in evolutionary adaptation. In *S*. Typhi, *sseJ *is considered a pseudogene. In this work, we studied the effect of *trans*-complementing *S*. Typhi with the *S*. Typhimurium *sseJ *gene and assessed the phenotype in human cell lines. Our results show that the presence of the *sseJ *gene induces phenotypic changes in cytotoxicity and in intracellular proliferation of *S*. Typhi in human epithelial cell lines. Our results suggest that the loss of SseJ function contributes to the development of a systemic infection in *S*. Typhi.

## Results

### *sseJ *is a pseudogene in *S*. Typhi

**Figure 1:**
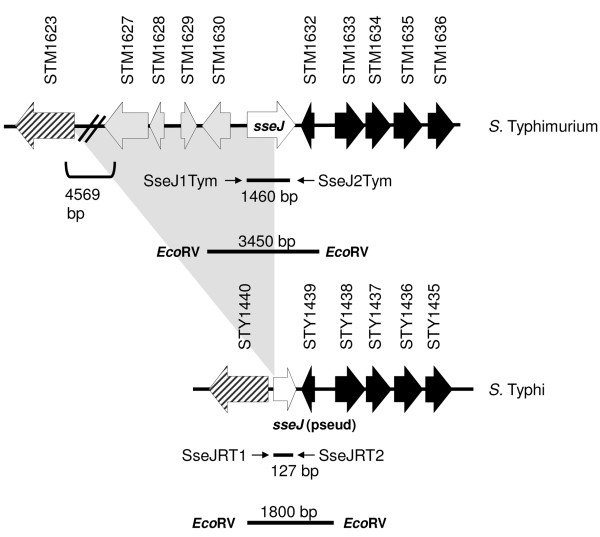
**Genomic organization of *sseJ *in *S*. Typhi and *S*. Typhimurium**. The figure shows the annealing localization of the primers designed (small arrows), the recognition sites of *Eco*RV and the *sseJ *probe hibridisation site (thick black line labelled 3450 bp for *S*. Typhimurium and 1800 bp for *S*. Typhi). The data were obtained from *S*. Typhi CT18 and *S*. Typhimurium LT2 genomes, available in public databases www.ncbi.nih.gov.

**Figure 2:**
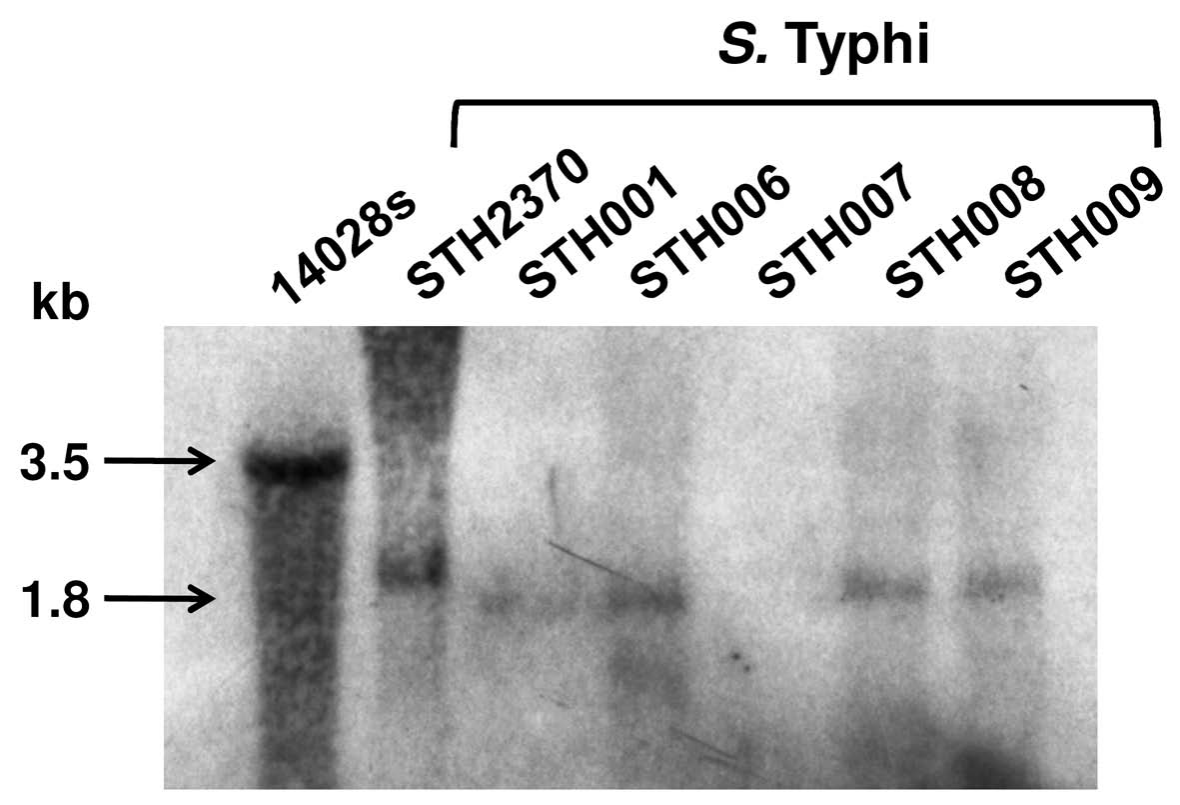
**Southern blot analysis of *sseJ *in *S*. Typhimurium and *S*. Typhi strain collection**. Genomic DNA digested with *Eco*RV was electrophoresed on an agarose gel and analyzed by Southern. Bands correspond to *S*. Typhimurium *sseJ *gene (3.5 Kb) or *S*. Typhi *sseJ *pseudogene (1.8 Kb).

To assess whether the *sseJ *locus is a pseudogene in the serovar Typhi, we compared the available sequences of *S*. Typhi Ty2, *S*. Typhi CT18 and *S*. Typhimurium LT2 [15,32,33]. We observed that the sequence corresponding to *sseJ *in *S*. Typhi is a 3' partial remnant of 141 bp, in contrast with the complete ORF found in *S*. Typhimurium (1227 bp). In order to corroborate these bioinformatics results, we designed a PCR assay with two sets of primers. The primers SseJ1Tym + SseJ2Tym yield a 1460 bp amplicon only when *sseJ *is complete, while the primers SseJRT1 + SseJRT2 yield a 127 bp amplicon if the 3' *sseJ *locus is present (Figure 1). As shown in Table 1 we observed a PCR product with the SseJRT1 + SseJRT2 primers in all the strains tested, including the reference strains (*S*. Typhi CT18, *S*. Typhi Ty2 and *S*. Typhimurium LT2) and *S*. Typhi clinical strains obtained from Chilean patients (STH collection). Nevertheless, we observed a PCR amplicon with the SseJ1Tym + SseJ2Tym primers only when the *S*. Typhimurium genomic DNA was used as template, strongly suggesting that the *sseJ *gene is an incomplete gene (i.e., a pseudogene) not only in the *S*. Typhi Ty2 and CT18 strains, but in all the Typhi clinical strains tested. To independently assess this hypothesis, we performed a Southern blot using the 1460 bp amplicon as a specific probe (Figure 2). The annealing of the probe with the *Eco*RV digested genome of *S*. Typhimurium yielded a 3450 bp fragment, while in *S*. Typhi, we observed a 1800 bp fragment. As shown in Figure 2 our data indicated that the presence of the pseudogene in *S*. Typhi CT18 is conserved in the *S*. Typhi clinical collection (STH). Therefore, the *sseJ *pseudogene seems to be a feature in serovar Typhi that distinguishes it from the serovar Typhimurium. *S*. Typhi STH007 presents no hibridisation with the probe, showing that this strain presents a larger deletion in the *sseJ *locus compared with other strains tested. *S*. Typhi STH2370 showed a slightly larger fragment than the other *S*. Typhi clinical strains presumably because of point mutations that changed the *Eco*RV restriction sites. Therefore, serovar Typhi has a genetic mutation in *sseJ *gene correlating with the previous studies made in strain CT18. We reasoned that the *sseJ *gene in the serovar Typhi is inactivated.

**Table 1:** PCR and Southern blot analysis of sseJ gene in S. Typhimurium vs. S. Typhi isolates

Strain	PCR 1460 bp	PCR 127 bp
**Strains**		
Serovar Typhimurium		
ATCC14028s	+	+
LT2	+	+
Serovar Typhi		
STH2370	-	+
STH001	-	+
STH004	-	+
STH005	-	+
STH006	-	+
STH007	-	+
STH008	-	+
STH009	-	+
Ty2	-	+

### *S*. Typhi harbouring the *S*. Typhimurium *sseJ *gene exhibits a decreased disruption of HT-29 polarised monolayers

**Figure 3:**
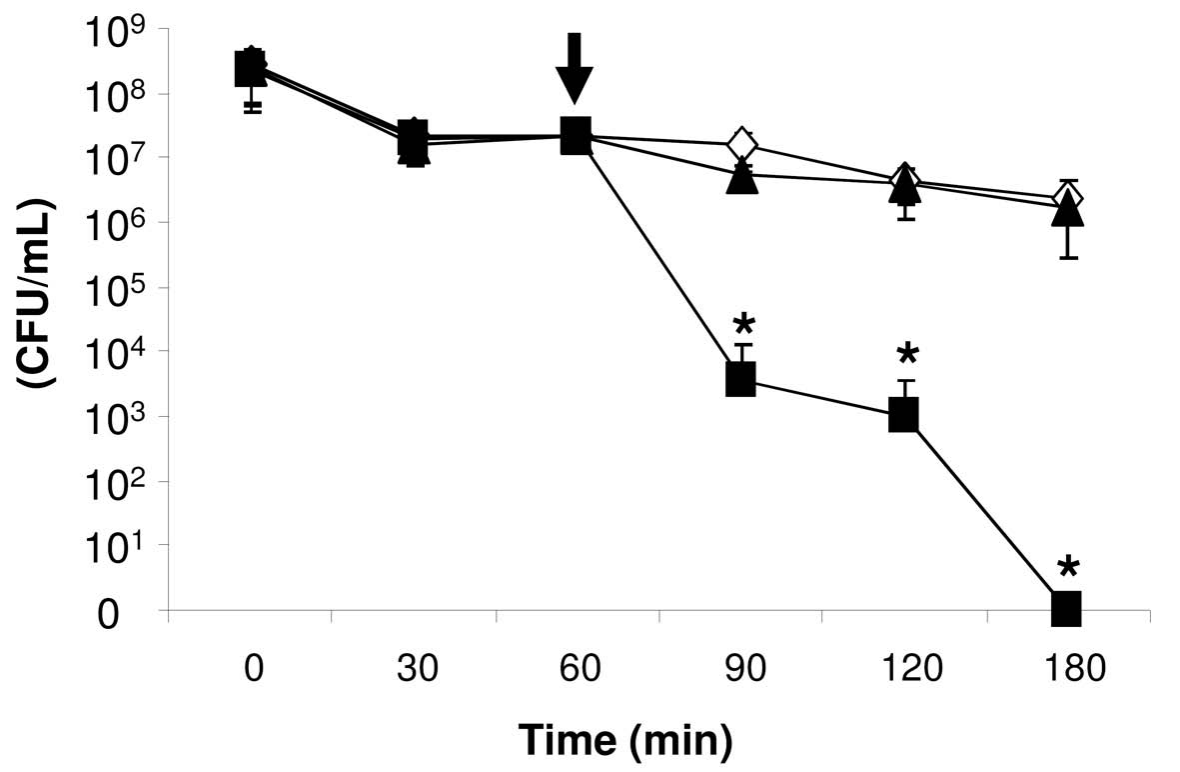
**Cell permeability assay of *S*. Typhi and *S*. Typhimurium through H-T29 human cell line monolayers**. (White diamonds) *S*. Typhimurium 14028s, (black squares) *S*. Typhi STH2370, (black triangles) *S*. Typhi STH2370/pNT005. The arrow indicates the time at which gentamicin was added. The results represent the average of three independent experiments. Each experiment was performed in duplicate. The values are expressed as the means ± SD of three independent experiments (asterisks represent p < 0.005). The CFU × ml^-1 ^numbers from infected cells with *S*. Typhi carrying empty plasmid (pSU19 or pCC1) showed no differences with respect to the wild type strain (data not shown).

If the loss of SseJ function in *S*. Typhi is advantageous for the interaction of bacteria with host cells, we should observe that wild type *S*. Typhi will present a different behaviour than the *S*. Typhi harbouring the *S*. Typhimurium *sseJ *gene when they are in contact with eukaryotic cells. This hypothesis was first tested by infecting polarised HT-29 monolayers with the strains under study using a modified transepithelial migration assay that included addition of gentamicin (after 1 h of infection, see Materials and Methods) into the upper chamber (black arrow, Figure 3). As shown in Figure 3 the recovered CFU × ml^-1 ^represented the bacteria which migrated to the lower chamber and survived the presence of the gentamicin that passed through the cell monolayer. If the integrity of the monolayer is disrupted by bacteria, gentamicin will leak through the lower chamber decreasing the recovered CFU × ml^-1^. If the monolayer is not disrupted, the recovered CFU × ml^-1 ^should remain essentially constant over the same time course. As observed in Figure 3 the recovered CFU × ml^-1 ^corresponding to *S*. Typhimurium 14028s presented a slight decline over the time course of the assay (white diamonds), suggesting that the monolayer integrity is not largely affected by bacteria. In contrast, the CFU × ml^-1 ^of *S*. Typhi STH2370 recovered from the lower chamber abruptly decreased until they became undetectable, strongly suggesting that the gentamicin leaked into the lower chamber due to a monolayer disruption (black squares). When *S*. Typhi were complemented with the *S*. Typhimurium *sseJ *gene (*sseJ*_STM_) (in the pNT005 plasmid, see Materials and Methods), and used to infect the monolayer, we observed that the corresponding recovered CFU × ml^-1 ^remained essentially constant, marking a sharp difference with the otherwise isogenic wild type strain and highly resembling the *S*. Typhimurium phenotype (compare the white diamonds and black triangles).

**Figure 4:**
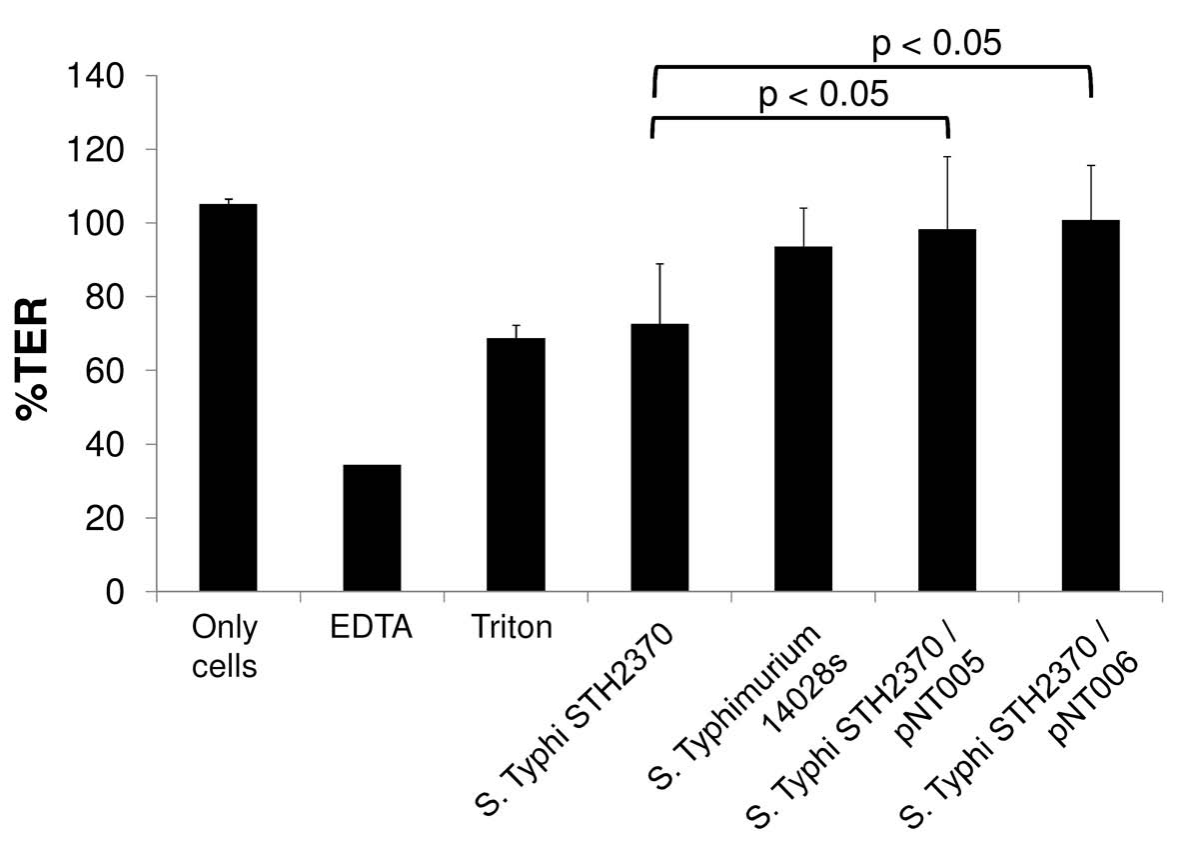
**The presence of the *sseJ *gene in *S*. Typhi promotes the disruption of the epithelial monolayer**. HT-29 cells were grown in transwells for 12-15 days. Polarised HT-29 cells were apically infected with the wild type *S*. Typhi or the respective complemented strains. TER 1 h post-infection reported as a percentage of the initial TER value and is expressed as the means ± SD of three different experiments, each performed in duplicate. The percentages of TER values from cells infected with *S*. Typhi carrying each empty plasmid (pSU19 or pCC1) showed no differences with respect the wild type strain (data not shown).

In order to independently assess whether *S*. Typhi harbouring the *S*. Typhimurium *sseJ *gene shows a decreased disruptive effect toward cultured cell monolayers than the wild type *S*. Typhi, we measured the transepithelial electrical resistance (TER). TER is a measure of the movement of ions across the paracellular pathway. Measurement of TER across cells grown on permeable membranes can provide an indirect assessment of tight junction establishment, stability and monolayer integrity [34]. As shown in Figure 4 after 1 h of infection wild type *S*. Typhi efficiently disrupted the monolayer as inferred by the lower TER measured compared with the control without bacteria. However, when HT-29 cells were infected with *S*. Typhi/pNT005, TER values were similar to those obtained with *S*. Typhimurium 14028s. This result indicates that *S*. Typhi/pNT005 was less disruptive on the monolayer than *S*. Typhi wild type, supporting the result shown in Figure 3. To discard a possible gene dosage effect by the vector copy number, we infected cells with *S*. Typhi/pNT006 (complemented with a single-copy vector harbouring *sseJ*_STM_) and the TER obtained was similar to that of *S*. Typhi/pNT005. This result demonstrated that the effect on cell permeability was due to the presence of s*seJ*_STM _and not to an artifact produced by gene dosage.

### *S*. Typhi harbouring *sseJ*_STM _was less cytotoxic than wild type *S*. Typhi

**Figure 5:**
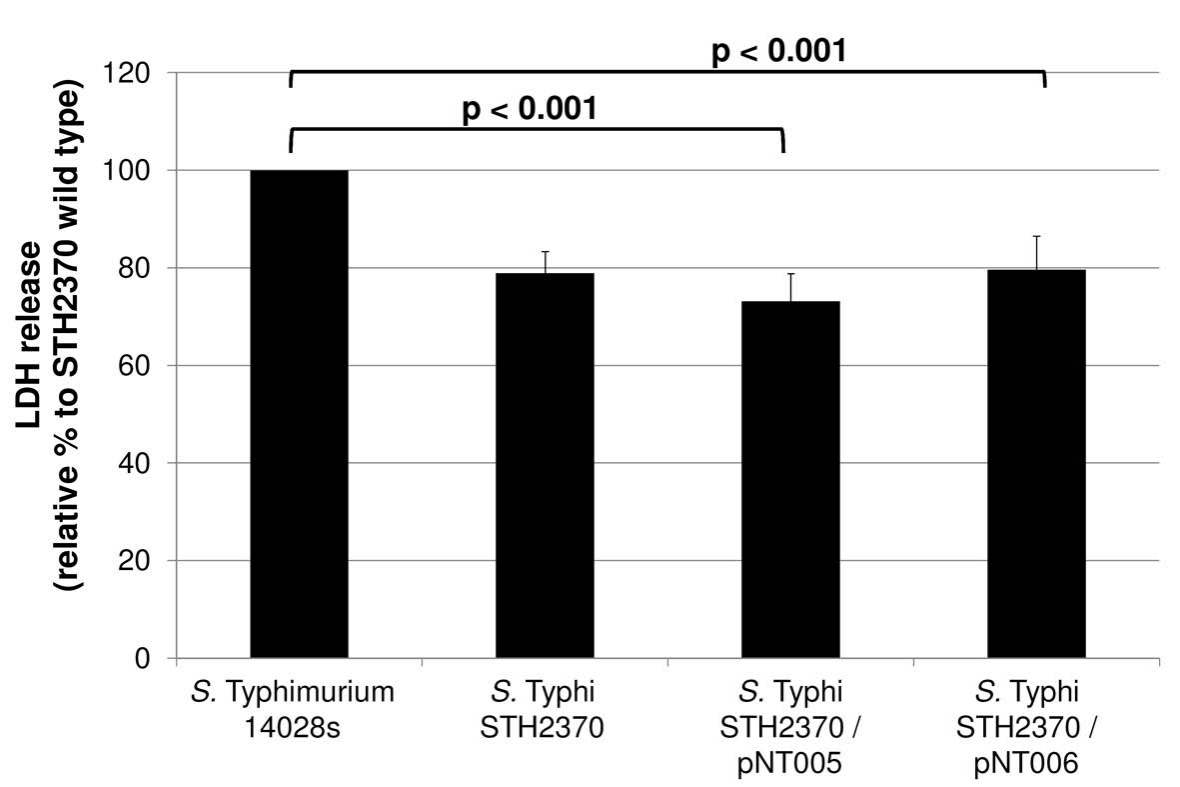
**Analyses of cytotoxicity HT-29 infected with complemented and wild type *S*. Typhi strains**. **H**T-29 cells were grown in transwells for 12-15 days. Polarised HT-29 cells were apically infected with the *S*. Typhi wild type or the respective complemented strains. Released LDH was measured 3 h post-infection and reported as percentage relative to the *S*. Typhi wild type. The values correspond to the means ± SD of three independent experiments, each performed in duplicate. The percentages of each *S*. Typhimurium 14028s, *S*. Typhi STH2370/pNT005 and *S*. Typhi STH2370/pNT006, have significantly differences respect *S*. Typhi STH2370 wild type. LDH release from infected cells with *S*. Typhi carrying empty plasmid (pSU19 or pCC1) showed no differences with respect to the wild type strain (data not shown).

Kops *et al*. demonstrated that *S*. Typhi Ty2 causes rapid death of some C2BBe cells in monolayers [35]. Because cell monolayer permeability may be increased due to cell death during infection, we wanted to assess whether the presence of s*seJ*_STM _in *S*. Typhi contributes to decrease cytotoxicity, as the results of the Figure 3 and 4 strongly suggest. Cell membrane damage due to cytotoxicity leads to the release of cytoplasmic enzymes, and the measurement of lactate dehydrogenase (LDH) release is a well-accepted assay to estimate cell membrane integrity and quantify cell cytotoxicity [36,37]. Then, the LDH release induced by *S*. Typhimurium, *S*. Typhi, *S*. Typhi/pNT005 or *S*. Typhi/pNT006 was compared. As shown in Figure 5 we found that wild type *S*. Typhi STH2370 was the most cytotoxic strain among all bacteria tested. This result suggests that the SseJ effector protein decreased *S*. Typhi cytoxicity when bacteria interact with human cell lines, resulting in increased cell permeability.

### The presence of *sseJ*_STM _in *S*. Typhi increased bacterial intracellular retention/proliferation within HEp-2 cells

**Figure 6:**
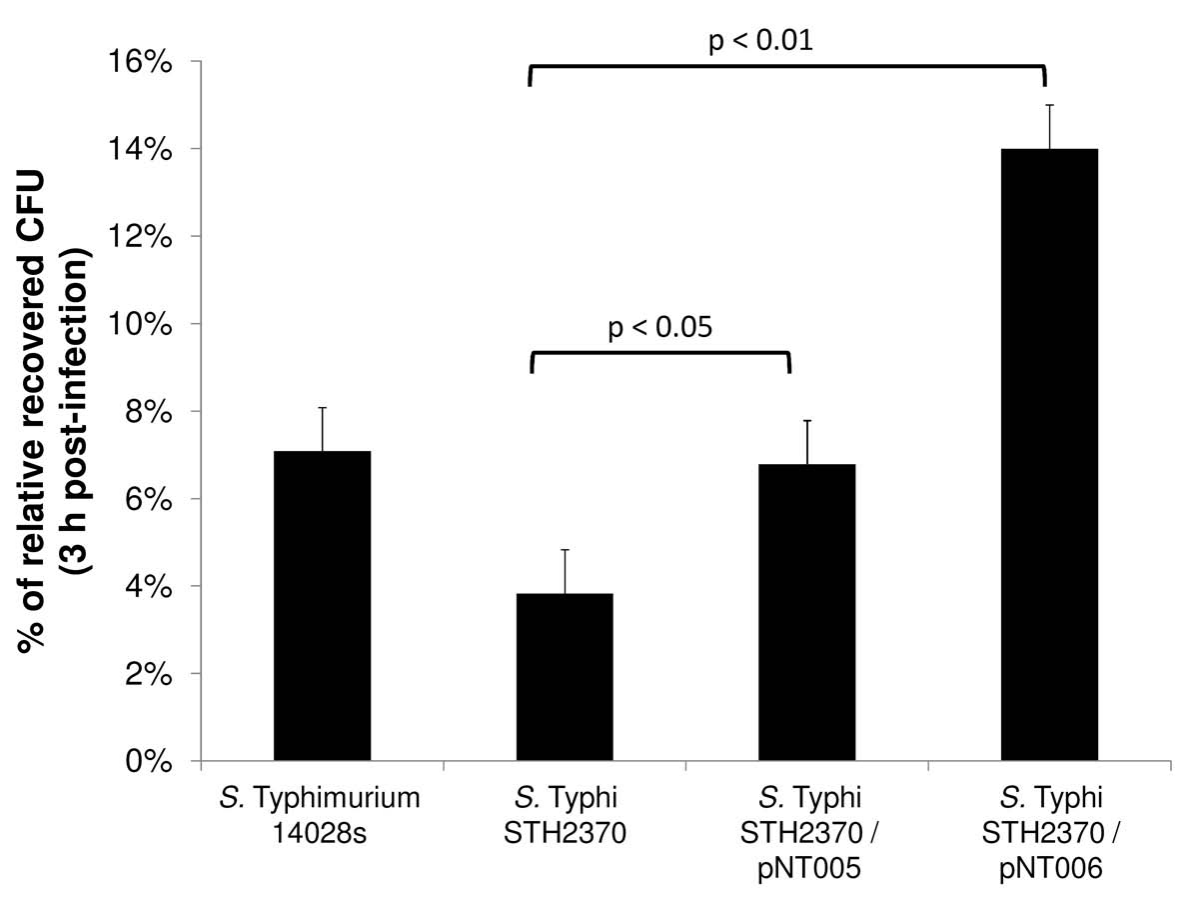
**Gentamicin protection assay of complemented and wild type strains of *S***. Typhi. HEp-2 cells were grown and infected with the *S*. Typhimurium 14028s, *S*. Typhi STH2370 or the respective *S*. Typhi complemented strains. The recovered CFUs were counted 3 h post-infection. The values correspond to the means ± SD of three different experiments, each performed in triplicate. The CFUs recovered from infected cells with *S*. Typhi with each empty plasmid (pSU19 or pCC1) showed no differences with respect to the wild type strain (data not shown).

It has been reported that *sseJ *contributes to the intracellular proliferation of *S*. Typhimurium [31,38]. Moreover, the decreased cell death produced by the presence of *sseJ*_STM _in *S*. Typhi strains (Figure 5) may lead to an increased proliferation of intracellular bacteria because of a decreased cytotoxicity. A less cytotoxic pathogen should be retained inside eukaryotic cells over time, allowing an increased bacterial proliferation. If this hypothesis is correct, *S*. Typhi carrying *sseJ*_STM _should exhibit increased CFUs in the gentamicin protection assay (see Materials and Methods). As expected, Figure 6 shows that the presence of *sseJ*_STM _yielded a significantly increase in the CFUs recovered from the infected cells compared to the wild type.

## Discussion

In the process of adaptation to humans, genes no longer compatible with the lifestyle of *S*. Typhi within the host were selectively inactivated. These inactivated genes are called "antivirulence genes" and their loss of function results in the adaptation to a given host [39]. *S*. Typhi is a facultative bacterial pathogen that has accumulated a high number of pseudogenes (approximately 5% of the genome) and over 75% of them have completely lost their functions [7,16]. When compared with the genome of free-living organisms, facultative pathogens harbour several pseudogenes and a population structure that promotes the maintenance of the mutations. In this context, *S*. Typhi represents an intermediate step between obligate bacterial parasites and free living bacteria, exhibiting some genome erosion directed to inactivate and lose detrimental or non-essential functions for their environment (i.e. host) [40]. Thus, we hypothesized that the loss of some of these genes contributed to the adaptation of *S*. Typhi to the systemic infection.

Our results suggest that the loss of the fully functional SseJ protein in *S*. Typhi contributed to the adaptation to the systemic infection by increasing bacterial cytotoxicity in epithelial cells. The increased cytotoxicity presented by *S*. Typhi compared with *S*. Typhimurium is not only related to the loss of functions, as we showed here with the *sseJ *pseudogene; but also to the acquisition of new functions. It has been reported that *S*. Typhi presents a pathogenicity island (named SPI-18) that harbours *hlyE*. The *hlyE *gene encodes a cytolysin that has proved to be cytotoxic toward different cell types [41-43]. SPI-18 is shared by other *Salmonella enterica *serovars that have been shown to cause systemic infections in humans, but is absent from *S*. Typhimurium [41]. In addition, the functional transfer of the *S*. Typhi *hlyE *gene to *S*. Typhimurium promotes deep organ infection in mice [41]. All this evidence suggests that *S*. Typhi has been selected for an increased cytotoxicity inside its host in order to perform a successful systemic infection. Thus, an increased cytotoxicity toward the epithelial barrier may guarantee the development of a deeper infection and a decreased retention inside epithelial cells at the bacterial entry point.

On the other hand, the presence of the *sseJ_STM _*gene in *S*. Typhi significantly enhances the retention time within epithelial cells and/or the intracellular proliferation as we showed in Figure 6 in agreement with previous reports that indicate that SseJ enzymatic activity

contributes to intracellular replication in host tissues [31,38]. Accordingly, it is possible that the *sseJ *loss of function was selected in *S*. Typhi in order to promote a decreased retention/proliferation of bacteria inside the eukaryotic cells. It is known that the intracellular proliferation is essential for the virulence of *S*. Typhimurium [44]. Nevertheless, recent studies revealed that the magnitude of the CD8^+ ^T cell response correlates directly to the intracellular proliferation in *Salmonella enterica*, showing that a reduced intracellular proliferation limits antigen presentation and development of a rapid CD8^+ ^T cell response, indicating that reduced intracellular proliferation of virulent pathogens may be an important mechanism of immune evasion. [45]. Accordingly, *Salmonella *presents several responses directed to downregulate the intracellular proliferation, reinforcing the concept that a state of low proliferation within the host cell is strategy to enhance virulence in a determined niche [46]. Actually, it has been shown that *Salmonella *expands its population in the liver by increasing the number of infection foci rather than undergoing massive intracellular growth in individual host cells, where the bacterial spreading from the initial infection foci to nearby cells may be facilitated by inducing cytotoxic effects in the infected cells [47,48].

How *sseJ_STM _*reduces the cytotoxicity in *S*. Typhi is not clear. It is known that the lipid imbalance associated to the presence of lipid alcohols, fatty acid and sterols is related to cytotoxicity and apoptosis [49,50]. Any process that limits the accumulation of these species is likely to be cytoprotective [50]. One such process involves the presence of different acyltransferase gene families that generate neutral lipids or steryl esters from these lipid alcohols [50]. SseJ, that presents glycerophospholipid: cholesterol acyltransferase (GCAT) activity in eukaryotic cells [51], might plausibly contribute to the reduction of the lipid-associated cytoxicity. The precise mechanisms underlying this process is unknown, but one possibility is that the presence of *sseJ_STM _*in *S*. Typhi is affecting the lipid remodelling in the infected cells, in turn reducing the cytotoxicity.

All our results together suggest that the loss of the *sseJ *gene in *S*. Typhi contributed to the adaptation to the systemic infection by increasing the bacterial-induced cytotoxicity and by decreasing the retention/proliferation inside the epithelial cells.

## Conclusions

Based on our results we conclude that the mutation that inactivate the *sseJ *gene in *S*. Typhi resulted in evident changes in the behaviour of bacteria in contact with eukaryotic cells, plausibly contributing to the *S*. Typhi adaptation to the systemic infection in humans.

## Methods

### Bacterial strains, media and growth conditions

The *S*. Typhi and *S*. Typhimurium strains used in this study are described in Table 2. Strains were routinely grown in Luria-Bertani (LB) medium (Bacto Tryptone 10 g × l^-1^; Bacto Yeast Extract 5 g × l^-1^, NaCl 5 g × l^-1^) at 37°C, with vigorous shaking, or anaerobically by adding an overlay of 500 μl of sterile mineral oil as a barrier to oxygen prior to invasion assays with cultured human cells. When required, the medium was supplemented with antibiotics at the following concentrations: chloramphenicol 20 μg × ml^-1^, ampicillin 100 μg × ml^-1 ^and kanamycin 50 μg × ml^-1^. Media were solidified by the addition of agar (15 g × l^-1 ^Bacto agar).

**Table 2:** Bacteria strains and plasmids used in this study

Strain or plasmid	Relevant characteristic	Reference or Source
**Strains**		
Serovar Typhimurium		
ATCC14028s	Wild-type strain, virulent	ATCC
LT2	Wild-type strain	S. Maloy
Serovar Typhi		
STH2370	Clinical strain, virulent	Hospital Dr Lucio Córdova
STH001	Clinical strain, virulent	Hospital Dr Lucio Córdova
STH004	Clinical strain, virulent	Hospital Dr Lucio Córdova
STH005	Clinical strain, virulent	Hospital Dr Lucio Córdova
STH006	Clinical strain, virulent	Hospital Dr Lucio Córdova
STH007	Clinical strain, virulent	Hospital Dr Lucio Córdova
STH008	Clinical strain, virulent	Hospital Dr Lucio Córdova
STH009	Clinical strain, virulent	Hospital Dr Lucio Córdova
Ty2	Wild-type strain	Instituto de Salud Pública
**Plasmids**		
pGEM-Teasy	High-copy-number cloning vector	Promega
pCC1	Single-copy vector, F plasmid derived	Stratagene
pNT002	pGEM-Teasy carrying the *S*. Typhimurium *sseJ *gene	This work
pSU19	Medium-copy-number cloning vector	[52]
pNT005	pSU19 carrying the *S*. Typhimurium *sseJ *gene	This work
pNT006	pCC1 carrying the *S*. Typhimurium *sseJ *gene	This work

### Construction of plasmids

The *sseJ *PCR product was initially cloned into pGEM-T Easy (Promega) to yield plasmid pNT002, and the presence of the gene was confirmed by PCR amplification and restriction endonuclease assays. The DNA fragment containing the *sseJ *gene was obtained from pNT002 and cloned into the *EcoRI *site of the medium-copy number vector pSU19 [52] to yield the plasmid pNT005. The presence of the gene and its promoter region was confirmed in all plasmids by PCR amplification and restriction endonuclease analyses. The PCR product was directly cloned in the pCC1 vector according to manufacturer's instructions (CopyControl™ PCR Cloning Kit, Stratagene) to yield the plasmid pNT006. The expression of *sseJ *gene from each plasmid was confirmed by Western blotting (data not shown).

### Bioinformatic analyses

Comparative sequence analyses were made with the complete genome sequences of *S. enterica *serovar Typhi strains CT18 (GenBank: AL627270.1) and Ty2 (GenBank: AL513382), serovar Typhimurium LT2 (GenBank: AE006468.1). The sequences were analyzed using the BLAST, alignment, and phylogeny tools available at http://www.ncbi.nlm.nih.gov/ and by visual inspection to improve alignments.

### PCR amplification

PCR amplifications were performed using an Eppendorf thermal cycler and *Taq *DNA polymerase (Invitrogen Cat. N° 11615-010). Reaction mixtures contained 1 × PCR buffer, 1.5 mM MgCl_2_, each dNTP (200 mM), primers (1 mM), 100 ng of template DNA, and 2 U polymerase. Standard conditions for amplification were 30 cycles at 94°C for 30 seconds, 62°C for 1 min and 72°C for 2 min 30 seconds, followed by a final extension step at 72°C for 10 min. Template *S*. Typhi chromosomal DNA was prepared as described [53]. Primers SseJ1Tym (CATTGTATGTATTTTATTGGCGACG) and SseJ2Tym (AATCGGCAGCAAAGATAGCA) were used to amplify 1460 bp, and were designed from the *S*. Typhimurium LT2 *sseJ *reported sequence. The conditions for amplification of 127 bp were 30 cycles at 94°C for 30 seconds, 53°C for 30 seconds and 72°C for 1 min, followed by a final extension step at 72°C for 10 min. Primers SseJRT1 (GCTAAAGACCCTCAGCTAGA) and SseJRT2 (CAGTGGAATAATGATGAGCT) were designed from the *S*. Typhimurium LT2 *sseJ *reported sequence.

### Southern hybridisations

Hybridisation probes for *sseJ *were generated by PCR amplification and were purified and labelled using the Detector™ Random Primer DNA Biotinylation Kit (KPL). Genomic DNA from *Salmonella *serovars was prepared as described by Maloy [54], cleaved with *EcoRV *(Invitrogen) and the fragments were resolved on a 0.8% agarose gel. The DNA was then transferred to a nylon membrane and cross-linked by UV irradiation. Hybridisation was performed according to the protocol described in the chemiluminescent system, using a DNA Detector™ HRP Southern Blotting Kit (KPL) and Kodak XAR-5 film.

### Cell permeability assay

We used an *in vitro *assay modified from the method described by McCormick [55]. Briefly, the colon carcinoma HT-29 cell line was grown to confluence (18-21 days) on 3.0 μm pore-size filters ("transwells", Millicell^®^, Millipore) with glucose-free RPMI (Gibco). Each transwell was inoculated individually to the apical surface with 400 μl of approximately 1 × 10^7 ^CFU ml^-1 ^of bacterial cultures and immediately incubated for 60 min at 37°C. After extensive washing with sterile PBS (NaCl 0.8% w/v; KCl 0.02% w/v; Na_2_HPO_4 _2H_2_O 0.13% w/v; KH_2_PO_4 _0.02% w/v), the extracellular bacteria were killed by treatment of monolayers with gentamicin (50 μg × ml^-1^). Immediately after gentamicin treatment, the medium from basal compartment of the epithelial cell monolayer was collected and plated for colony forming units (CFU) to assess the number of bacteria that passed through the cell monolayer. The polarisation of cells was confirmed by transepithelial electrical resistance (TER) and transmission electron microscopy (data not shown).

### Transepithelial electrical resistance

TER was used to monitor changes in epithelial cell culture integrity. TER in HT-29 enterocytes was studied using an EVOM electrode (World Precision Instruments). The enterocytes were grown to confluence (18-21 days) on 3.0 μm pore-size filters ("transwells", Millicell^®^, Millipore). The electrical resistance readings were recorded after subtracting the average resistance of two membranes in the absence of enterocytes at the beginning of the assay (t_0_) and 1 h post-infection (t_1_). Controls included the incubation of the cells with EDTA and Triton X-100 (1% PBS). The reading was expressed as percentages and calculated as follows:

$$\%\text{TER}\left(1\text{h}\right)=100\times \left(\text{TER}\;{\text{t}}_{0}\times {\text{TER t}}_{1}{}^{-1}\right)$$

We verified the HT-29 polarisation by TER and transmission electron microscopy.

### LDH Cytotoxicity Assay

Cytotoxicity of infected HT-29 cells was assayed using a lactate dehydrogenase (LDH) Kit (Valtek), which measures the extracellular release of LDH into the media by dead cells, according to the manufacturer's instructions. The absorbance values of treated cells were expressed as a percentage relative to the wild type *S*. Typhi after correcting for background from media without cells at 340 nm.

### Gentamicin protection Assay

To measure bacterial invasion, the method described by Lissner [56] and modified by Contreras [57] was used. Briefly, HEp-2 monolayers (5 × 10^5 ^cells/well) were grown at 37°C in a 5% CO_2_/95% air mixture in RPMIFS (RPMI medium supplemented with 10% fetal bovine serum pre-treated for 30 min at 60°C). The tested bacterial strains were grown anaerobically to mid-exponential phase and then harvested by centrifugation prior to infect the monolayers in 96-well microtiter plates at a multiplicity of infection of 100:1. After incubation of 1 h to allow bacterial entry into the cells, monolayers were washed twice with phosphate-buffered saline (PBS), and 100 μL of RPMI containing gentamicin (200 μg × ml^-1^) was added to each well. The plates were then incubated for 2 h to kill any remaining extracellular bacteria. In the case of the strains carrying vectors, the medium was supplemented additionally with chloramphenicol during the entire assay. The medium was removed and cells were washed twice with PBS. Then, the cells were lysed with sodium deoxycholate (0.5% w/v, in PBS). The number of intracellular bacteria (CFU at t_3_) was determined plating onto LB agar plates with chloramphenicol (the strains carrying plasmid) or without antibiotic (the wild type strains). Quantitative invasion assay values were calculated as follows:

$$3\;\text{h}\;\text{post}\;\text{infection}\;\text{index}=100\times \left(\text{intracellular}\;\text{CFU}\;{\text{mL}}^{-1}\text{at}\;{\text{t}}_{3}\times \text{CFU}\;{\text{mL}}^{-1}\text{added}\right)$$

### Statistics

All results are expressed as means ± SD of an individual experiment performed in triplicate. *P *values were calculated according to Student's *t-*test, and values p < 0.05 or p < 0.01 were considered statistically significant.

## Authors' contributions

AT: designed the studies, performed the experiments and wrote the manuscript; LB: performed the transepithelial electrical resistance experiment, contributing significantly in the development of the other experiments and in the preparation of manuscript; JAF: participated in writing the paper; GCM: designed the studies and participated in the revision of the manuscript. All authors read and approved the final manuscript.
